# Comparative mitochondrial genomics of* Thelebolaceae* in Antarctica: insights into their extremophilic adaptations and evolutionary dynamics

**DOI:** 10.1186/s43008-024-00164-7

**Published:** 2024-10-30

**Authors:** Zechen Mi, Jing Su, Liyan Yu, Tao Zhang

**Affiliations:** China Pharmaceutical Culture Collection, Institute of Medicinal Biotechnology, Chinese Academy of Medical Sciences & Peking Union Medical College, Beijing, 100050 People’s Republic of China

**Keywords:** Psychrophiles, Mitogenome, Phylogeny, *Thelebolus*, *Antarctomyces*, *Leotiomycetes*

## Abstract

Species of *Antarctomyces* and *Thelebolus* (*Thelebolaceae*), primarily found in Antarctic environments, exhibit psychrophilic adaptations, yet their mitochondrial genomes have not been extensively studied. Furthermore, few studies have compared the mitochondrial genomes of psychrophilic, psychrotrophic, and mesophilic fungi. After successful sequencing and assembly, this study annotated the mitochondrial genomes of *Antarctomyces psychrotrophicus* CPCC 401038 and *Thelebolus microsporus* CPCC 401041. We also performed a comparative analysis with the previously characterized mitochondrial genomes of psychrotrophic and mesophilic fungi. The analysis revealed that *nad4L* was the most conserved gene across the mitochondrial genomes, characterized by its synonymous and non-synonymous substitution rates (Ks and Ka), genetic distance, and GC content and skew within the protein-coding genes (PCGs). Additionally, the mitochondrial genomes of psychrophilic and psychrotrophic fungi showed a higher proportion of protein-coding regions and a lower GC content compared to those of mesophilic fungi, underscoring the genetic basis of cold adaptation. Phylogenetic analyses based on these mitochondrial genes also confirmed the phylogenetic relationships of *Thelebolaceae* in the class *Leotiomycetes*. These findings advance our understanding of the phylogenetic relationships and evolutionary dynamics within the family *Thelebolaceae*, highlighting how different environmental temperatures influence fungal mitochondrial genomic structure and adaptation.

**Supplementary Information:**

The online version contains supplementary material available at 10.1186/s43008-024-00164-7.

## Introduction

Antarctica, known as the world’s harshest environment, contains nearly 90% of the planet's ice (Kennicutt et al. 2014) and has about 35% of its land surface covered by snow (Margesin et al. 2011). It experiences sub-zero temperatures, seasonal freeze–thaw cycles, intense UV radiation, and arid conditions (Batista et al. 2020; de Menezes et al. 2017; Ordóñez-Enireb et al. 2022). Its unique environmental conditions surpass those of other continents, facilitating the growth of diverse species and providing excellent opportunities to study life under extreme conditions (Batista et al. 2020). Despite the harsh conditions, microbiota—including bacteria, archaea, and fungi—constitute the majority of the biomass and dominate Antarctic ecology (Pointing et al. 2009; Arenz et al. 2006). The ability of fungi to colonize and persist in diverse Antarctic environments and substrates highlights their significance as eukaryotic organisms (de Menezes et al. 2019). The majority of Antarctic fungi belong to the phylum *Ascomycota*, with *Basidiomycota*, *Mortierellomycota*, and *Chytridiomycota* also significantly present (Bridge et al. 2012; Rosa et al. 2019). Antarctic fungi display remarkable genetic, metabolic, and morphological adaptations that enable their survival, reproduction, and dispersion across diverse environmental conditions (Gomes et al. 2018).

In the genus *Antarctomyces*, only two species, *A. psychrotrophicus* and *A. pellizariae*, have been identified, both endemic to Antarctica (de Menezes et al. 2017; Stchigel et al. 2001). These species demonstrate psychrophilic traits, indicating their potential as sources of antifreeze compounds (Xiao et al. 2010; de Menezes et al. 2017). In contrast, species from the genus *Thelebolus* have been isolated from diverse extreme habitats, including Antarctic lakes and mosses (de Hoog et al. 2005; Zhang et al. 2013), Arctic lichens (Zhang et al. 2016), and Atlantic sponges (Bovio et al. 2018). Not all *Thelebolus* species are psychrotrophic or psychrophilic. Wicklow and Malloch (1971) studied the temperature adaptations of different *Thelebolus* species, showing different optimal growth temperatures at 15 °C, 20 °C, or 25 °C. Both genera, *Antarctomyces* and *Thelebolus*, belong to the family *Thelebolaceae*, indicating a shared evolutionary background but distinct ecological niches.

Mitochondria, vital organelles in eukaryotes, supply almost all the energy required for cellular activities (Muñoz-Gómez et al. 2017). Located in the cytoplasm, mitochondria also play roles in cellular processes, including information transfer, cell differentiation, senescence, and apoptosis (Burki 2016). Owing to its maternal inheritance, rapid evolution, and abundance of molecular markers, the mitochondrial genome is used more frequently in phylogenetic and evolutionary studies than the nuclear genome (Burger et al. 2003; Li et al. 2018a, b, c). Additionally, the mitochondrial genomes of various eukaryotes have evolved in terms of genome size, gene content, and base composition (Sayadi et al. 2017). Understanding the organization of mitochondrial genes and the structure of tRNAs can aid in uncovering evolutionary links between species.

Recent classifications place the family *Thelebolaceae* within the order *Thelebolales* in the class *Leotiomycetes*. Species in *Thelebolales* often inhabit extreme environments and exhibit specific adaptations (Quijada et al. 2022). However, the mitochondrial genomes of these species remain largely unexplored, with the NCBI database listing only one such genome, that of *Antarctomyces pellizariae*. In this study, we assembled and annotated the complete mitochondrial genomes of *A*. *psychrotrophicus* and *T. microsporus.* We analyzed and compared these mitochondrial genomes, highlighting both differences and similarities in their structure, gene content, and gene order. Additionally, considering the unique conditions of the polar environment, we compared the mitochondrial genome characteristics and gene rearrangements among fungi growing at various temperatures, including psychrophilic, psychrotrophic, and mesophilic species. The mitochondrial genomes of *A. psychrotrophicus* CPCC 401038 and *T. microsporus* CPCC 401041 have enhanced our understanding of the population genetics, taxonomy, and evolutionary biology of these genera. Furthermore, we found evidence of how temperature affects fungal development at the mitochondrial level.

## Materials and methods

### Sampling, DNA extraction, and sequencing

*Antarctomyces psychrotrophicus* CPCC 401038 and *Thelebolus microsporus* CPCC 401041, isolated from soil samples in the Fildes Region of maritime Antarctica, are preserved at the China Pharmaceutical Culture Collection (CPCC). The strains were cultured in potato dextrose broth at 15 °C and their mycelia were collected after 10 days. Genomic DNA was extracted using a fungal DNA kit (item D3390-00, Omega Bio-Tek, GA, USA) according to the manufacturer's instructions. The purified genomic DNA was quantified using a TBS-380 fluorometer (Turner BioSystems Inc., CA, USA). A sequencing library was constructed using the NEXTflex Rapid DNA-Seq Kit (Bioo Scientific, TX, USA), following the manufacturer's instructions. The prepared libraries were sequenced with paired-end sequencing (2 × 150 bp) on an Illumina HiSeq X Ten platform (Illumina Inc., CA, USA).

### Assembly of mitochondrial genomes

The assembly of mitochondrial genomes was performed through a multi-step approach. First, quality-controlled clean data were generated using fastp (https://github.com/OpenGene/fastp), which filtered out low-quality reads. The filtering criteria included removing reads with more than 5% N bases, reads with over 50% of the bases having a quality score of ≤ 5, and reads contaminated with adapters. The quality-controlled data were subsequently assembled using SPAdes v3.11.0 (Bankevich et al. 2012) with default parameters, generating a comprehensive set of scaffolds. These scaffolds were further filtered by aligning them using BLASTn and Exonerate against closely related mitochondrial sequences with the following thresholds: an e-value of 1e^−10^ for nucleotide sequences and 70% similarity for protein sequences. Only scaffolds with confirmed gene matches were retained for further analysis. Next, fragmented sequences were extended and merged through 50 iterations of PRICE (Paired-Read Iterative Contig Extension) (Ruby et al. 2013) and MITObim (Hahn et al. 2013) to reduce the number of scaffolds. To refine these assemblies, the original sequencing reads were realigned to the assembly using Bowtie2, then reassembled with SPAdes. During this refinement process, the assembly results were optimized using VelvetOptimiser to adjust the k-mer settings, with k-mer values set at 93, 95, 97, 103, 105, 107, and 115 for successive rounds of reassembly. The refined assembly was inspected for circularity. If no circular genome structure was detected, the extension and refinement processes were repeated iteratively until successful.

### Annotation of mitochondrial genomes

The two mitochondrial genomes were initially annotated by MITOS2 (Donath et al. 2019), MFANNOT (https://megasun.bch.umontreal.ca/apps/mfannot/), and tRNAscan-SE v2.0 (Lowe and Chan 2016), all applying the Genetic code 4. Protein-coding genes (PCGs), rRNA genes, and tRNA genes were annotated during this step and manually checked. The initial PCGs annotations were subsequently refined and adjusted using the NCBI Open Reading Frame Finder (https://www.ncbi.nlm.nih.gov/orffinder/) and further proofread via a BLASTP search against the NCBI Non-Redundant Protein Sequence Database. The complete mitochondrial genome was visualized and mapped using Proksee (https://proksee.ca) (Grant et al. 2023).

### Mitochondrial genome analysis across temperature-adapted fungi

To investigate the mitochondrial genome-level similarities and differences among fungi with various temperature adaptations, we selected three mesophilic fungi (*Blumeria graminis* MT880591, *Erysiphe necator* NC_056146, and *Monilinia fructicola* NC_056195), two psychrotrophic fungi (*Pseudogymnoascus destructans* NC_033907 and *Pseudogymnoascus pannorum* NC_027422), and three psychrophilic fungi (*A. pellizariae* NC_048507, *A. psychrotrophicus* CPCC 401038, and *T. microsporus* CPCC 401041). These three fungal groups all belong to the same class, *Leotiomycetes*, but differ in their growth temperature adaptations. Both psychrophilic and psychrotrophic fungi can grow at 0 °C. Psychrotrophic fungi have a maximum growth temperature above 20 °C, whereas psychrophilic fungi have a maximum growth temperature of 15 °C or below (Gounot 1986; Robinson 2001). Mesophilic fungi grow at temperatures ranging from 5 to 35 °C, with optimal growth occurring between 25 and 30 °C (Dix and Webster 1995).

The base composition of the mitochondrial genomes was analyzed using the Sequence Manipulation Suite (Stothard 2000), and strand asymmetry was evaluated using the following equations: AT skew = [A − T]/[A + T] and GC skew = [G − C]/[G + C] (Wang et al. 2017). Codon usage in the mitochondrial genomes was also analyzed using the Sequence Manipulation Suite, based on genetic code 4. Synonymous (Ks) and non-synonymous (Ka) substitution rates for PCGs in mitochondrial genomes was calculated using DnaSP v6.12.03 (Rozas et al. 2017). MEGA v11 (Tamura et al. 2021) was used to calculate the overall average genetic distances between each pair of the 14 core PCGs (*atp6*, *atp8*, *atp9*, *cox1*, *cox2*, *cox3*, *nad1*, *nad2*, *nad3*, *nad4*, *nad4L*, *nad5*, *nad6*, and *cob*) and *rps3*, using the Kimura-2-parameter (K2P) substitution model. Lastly, homologous segments of these mitochondrial genomes were analyzed using Mauve v2.4.0 (Darling et al. 2004).

### Phylogenetic analysis of mitochondrial genomes

To determine the phylogenetic relationships of *A. psychrotrophicus* CPCC 401038 and *T. microsporus* CPCC 401041, the complete mitochondrial genomes of 19 other species in the class *Leotiomycetes* were downloaded from the GenBank database. During the construction of the phylogenetic tree, several species, such as *Blumeria graminis*, lacked annotation for the gene encoding ATP synthase subunit 9 (*atp9*). Consequently, the phylogenetic tree was constructed by combining the 13 core PCGs (*atp6*, *atp8*, *cox1*, *cox2*, *cox3*, *nad1*, *nad2*, *nad3*, *nad4*, *nad4L*, *nad5*, *nad6*, and *cob*) along with *rps3* into a mitochondrial gene set. Genes from the mitochondrial genomes were extracted using PhyloSuite v1.2.3 (Xiang et al. 2023), and individual genes were aligned using MAFFT v7.037 (Rozewicki et al. 2019). MrModeltest2 (Nylander 2004) was used to determine the evolutionary model that best fits the combined data. A phylogenetic tree was subsequently constructed using MrBayes v3.2. 6 (Ronquist et al. 2012) with a Bayesian inference (BI) approach. In parallel, two independent runs with four chains—three heated and one cold—were performed, each running for 2 × 10^6^ generations. Every 100 generations, a run sample was collected. To evaluate node support, bootstrap (BS) values were produced using 1000 iterations for the ML analyses using RAxML v8. 2. 10 (Stamatakis 2014).

### Data availability

The following accession numbers for two newly sequenced fungal mitochondrial genomes are available in the GenBank database: *Antarctomyces psychrotrophicus* CPCC 401038: NC_082276; *Thelebolus microsporus* CPCC 401041: NC_082275.

## Results

### Characterization of the mitochondrial genomes of *A. psychrotrophicus* and *T. microsporus*

**Fig. 1:**
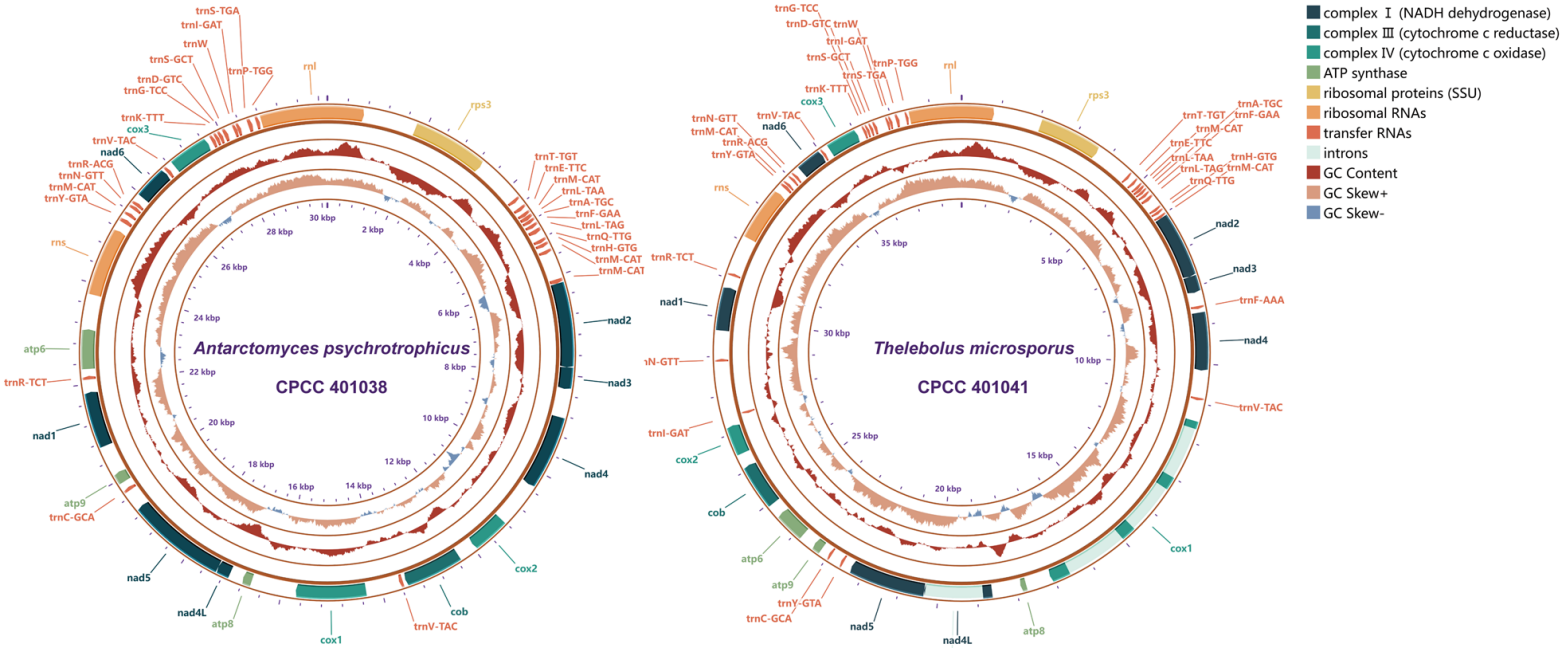
Mitochondrial genome circular maps of *A. psychrotrophicus* CPCC 401038 and *T. microsporus* CPCC 401041. Different genes are represented by distinct color blocks, with the outermost ring indicating the positive strand of the mitochondrial genome

The complete mitochondrial genomes of *A. psychrotrophicus* CPCC 401038 and *T. microsporus* CPCC 401041 are both circular DNA molecules, with genome sizes of 30,170 bp and 38,803 bp, respectively (Fig. 1). The GC content of *A. psychrotrophicus* CPCC 401038 is 29.62%, which is similar to that of *T. microsporus* CPCC 401041 (29.65%) (Table 1). Both *A. psychrotrophicus* CPCC 401038 and *T. microsporus* CPCC 401041 have negative AT skew (− 0.07, − 0.04) and positive CG skew (0.09, 0.11). A total of fifteen PCGs are identified in both mitochondrial genomes, including 14 genes coding for proteins involved in oxidative phosphorylation: seven subunits of the NADH dehydrogenase electron transport complex I (*nad1*, *nad2*, *nad3*, *nad4*, *nad4L*, *nad5*, and *nad6*), one subunit of complex III (*cob*), three subunits of complex IV (*cox1*, *cox2* and *cox3*), and three subunits of the ATP synthase complex (*atp6*, *atp8* and *atp9*). Additionally, the gene encoding the ribosomal protein *rps3* was annotated. Four introns, ranging in length from 1280 to 1588 bp, were identified in the mitochondrial genome of *T. microsporus* CPCC 401041, located within the genes *cox1* and *nad4L*. In contrast, no introns were detected in the PCGs of the *A. psychrotrophicus* CPCC 401038 mitochondrial genome. Except for differences in gene distribution, the PCGs of *A. psychrotrophicus* CPCC 401038 and* T. microsporus* CPCC 401041 were similarly annotated. Intergenic sequences of 10,207 bp and 12,783 bp were identified in the two mitochondrial genomes of *A. psychrotrophicus* CPCC 401038 and* T. microsporus* CPCC 401041, respectively. In the mitochondrial genome of *T. microsporus* CPCC 401041, intergenic sequences ranged in length from 0 to 1402 bp, with the longest intergenic sequence located between the tRNA genes *trnN-GTT* and *trnI-GAT*. The longest intergenic sequence in the *A. psychrotrophicus* CPCC 401038 mitochondrial genome was located between *rnl* and *rps3*. It is noteworthy that *nad4L* and *nad5* share an overlapping base and that *nad2* and *nad3* are closely linked in the mitochondrial genomes. Two rRNA genes, one encoding the large subunit ribosomal RNA (*rnl*) and one encoding the small subunit ribosomal RNA (*rns*), were present in each mitochondrial genome. The rRNA gene lengths differ between the two mitochondrial genomes, with a 107 bp difference in *rnl* and a 22 bp difference in *rns*.

**Table 1:** Comparison of mitochondrial genomic features in psychrophilic, psychrotrophic, and mesophilic fungi

Temperature adaptation	Species	Mitogenome size (bp)	GC%	AT%	GC skew	AT skew
Psychrophilic	*Antarctomyces pellizariae*	30,121	29.50	70.50	0.09	− 0.07
	*A. psychrotrophicus* CPCC 401038	30,170	29.62	70.38	0.09	− 0.07
	*T. microsporus* CPCC 401041	38,803	29.65	70.35	0.11	− 0.04
Psychrotrophic	*Pseudogymnoascus destructans*	32,181	28.53	71.47	0.12	− 0.05
	*Pseudogymnoascus pannorum*	26,918	28.10	71.90	0.11	− 0.06
Mesophilic	*Blumeria graminis*	109,800	48.28	51.72	0.17	0.02
	*Erysiphe necator*	188,577	33.86	66.14	0.10	0.03
	*Monilinia fructicola*	159,648	30.94	69.06	0.10	0.02

In both mitogenomes, the protein-coding regions constituted the majority, accounting for 37.43–48.03% of the total length (Fig. S1). The intergenic regions, comprising 33.83% and 32.94% of the total length of the two mitochondrial genomes, respectively, represented the second-largest portions. The intronic region accounted for 14.66% of the mitochondrial genomes of *T*. *microsporus* CPCC 401041, whereas only one tRNA gene with 11 bp of introns was found in *A*. *psychrotrophicus* CPCC 401038. Compared to *A*. *psychrotrophicus* CPCC 401038, the mitochondrial genome of *T. microsporus* CPCC 401041 was 8633 bp longer, with nearly identical numbers of bases in the RNA-coding and protein-coding regions in both genomes.

The mitochondrial genome of *A. psychrotrophicus* CPCC 401038 contains 27 tRNA genes, whereas that of *T. microsporus* CPCC 401041 contains 30 tRNA genes, all folded into a typical cloverleaf-type structure. The tRNA genes from *A. psychrotrophicus* CPCC 401038 and *T. microsporus* CPCC 401041 range in length from 71 to 86 bases. One of the tRNA genes encoding methionine in *A. psychrotrophicus* CPCC 401038 contains an 11 bp intron. In terms of tRNA gene distribution, those in *A. psychrotrophicus* CPCC 401038 are more concentrated, mainly between the two ribosomal RNA genes and between *nad2* and *rps3*. In contrast, the tRNA genes in the mitochondrial genome of *T. microsporus* CPCC 401041 are more scattered and distributed throughout the loop, with a copy of *trnI* containing the anticodon GAT located on the negative strand.

**Fig. 2:**
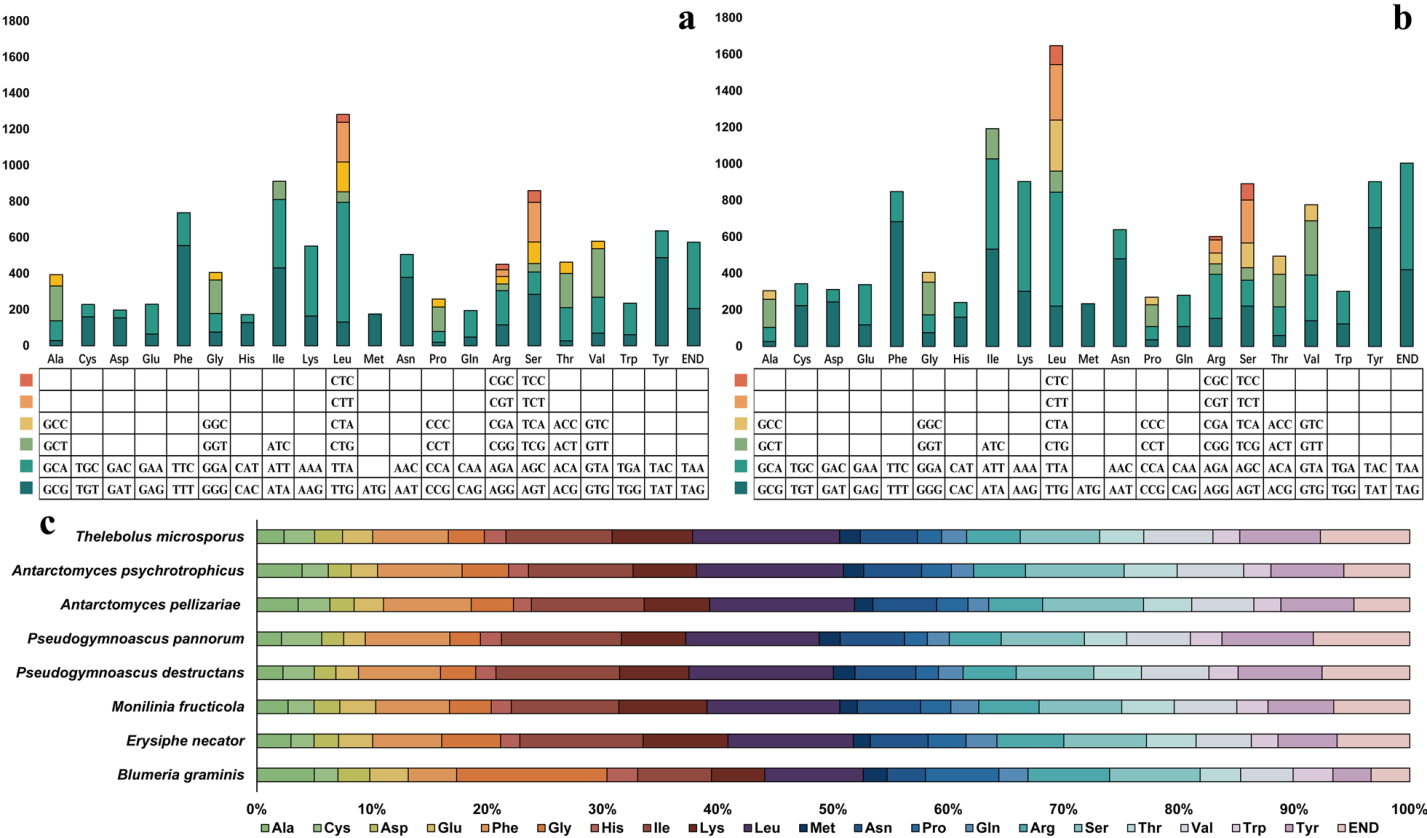
Comparative analysis of codon usage in the mitochondrial genomes of *A. psychrotrophicus* CPCC 401038 and *T. microsporus* CPCC 401041, along with amino acid usage in eight mitochondrial genomes. **a** codon usage of *A. psychrotrophicus* CPCC 401038; **b** codon usage of *T. microsporus* CPCC 401041; **c** amino acid usage in eight mitochondrial genomes

Codon usage analysis revealed that the most frequently used codons in the two mitochondrial genomes were TTA (leucine), TTT (phenylalanine), TAT (tyrosine), ATA (isoleucine), ATT (lysine), and AAA (lysine) (Fig. 2). The high AT content in the two mitochondrial genomes primarily results from the frequent use of A and T in codons. Amino acid usage analysis shows that both mitochondrial genomes have nearly the same amount of glycine, but *A. psychrotrophicus* CPCC 401038 uses significantly more alanine than *T. microsporus* CPCC 401041, a pattern that contrasts with the usage of other amino acids.

### Comparison of mitochondrial genomic characteristics among psychrophilic, psychrotrophic, and mesophilic fungi

The mitochondrial genomes of mesophilic fungi were approximately 3–6 times larger than those of psychrotrophic and psychrophilic fungi (Table 1). Additionally, their GC contents were higher. For example, the GC content of *B. graminis* was 48.28%, significantly higher than that of the five psychrotrophic and psychrophilic fungal strains. GC skew was consistently positive across these eight fungi, while AT skew varied. Three mesophilic fungi exhibited a positive AT skew, whereas the five psychrotrophic and psychrophilic fungi displayed a negative AT skew.

**Fig. 3:**
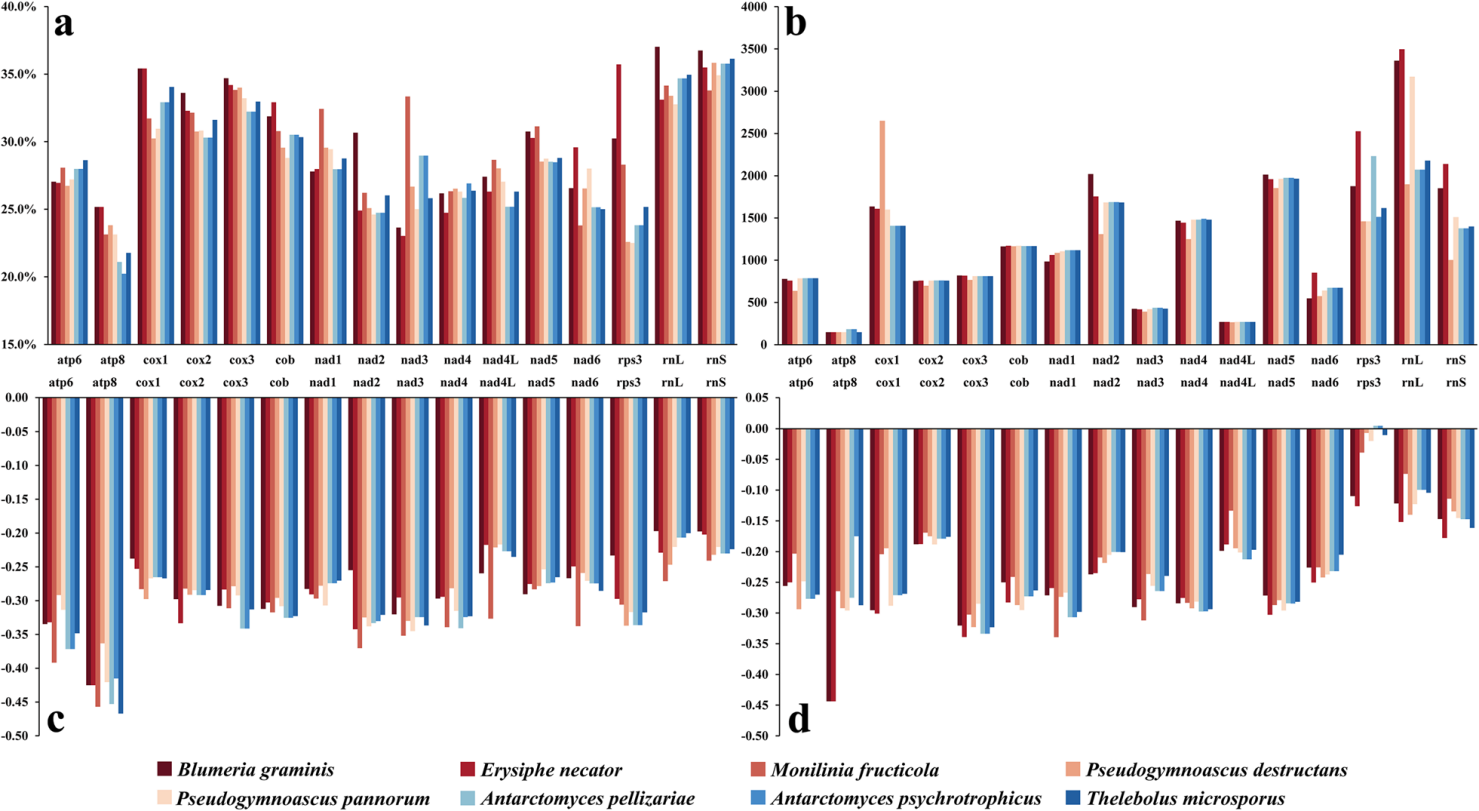
Comparison of PCG characteristics in the mitochondrial genomes of psychrophilic, psychrotrophic, and mesophilic fungi. **a** GC content; **b** gene length (bp); **c** GC skew; **d** AT skew

A comparison of the GC content of each gene revealed that the GC content of several genes, including *atp8*, *cox1*, *cox2*, c*ox3*, *cob*, *nad5*, and *rps3*, was higher in mesophilic fungi than in psychrotrophic and psychrophilic fungi (Fig. 3). Except for *E. necator*, the highest GC content in the other seven fungi was observed in the rRNA genes. When PCGs and rRNA genes were considered as a combined gene set for GC content comparison, mesophilic fungi exhibited slightly higher GC contents than psychrotrophic and psychrophilic fungi. The lengths of protein-coding regions were similar across psychrophilic, psychrotrophic, and mesophilic fungi. The length of tRNA coding region positively correlated with the number of tRNA genes in the mitochondrial genomes, whereas the rRNA coding region was longer in mesophilic fungi. The mitochondrial genomes of mesophilic fungi contained more non-coding regions, such as introns and intergenic sequences, which primarily accounted for their larger size. Additionally, in the five psychrotrophic and psychrophilic fungi, the proportion of protein-coding regions in the mitochondrial genomes was higher, whereas mesophilic fungi had a higher proportion of intergenic regions and introns (Fig. S2). The frequency of amino acid usage in each fungal strain was analyzed, revealing the high usage of leucine and isoleucine, and the lowest usage of methionine and histidine (Fig. 2).

### Gene rearrangement and collinearity analysis

**Fig. 4:**
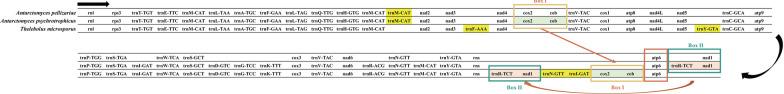
Comparison of gene arrangements in the mitochondrial genomes of *A. pellizariae*, *A. psychrotrophicus,* and *T. microsporus.* Genes with the yellow background represent tRNA genes unique to each mitochondrial genome. These highlighted genes indicate differences among the three mitochondrial genomes of the genera *Antarctomyces* and *Thelebolus*

Among the 15 PCGs detected in the mitochondrial genomes of *A. psychrotrophicus* CPCC 401038 and *T. microsporus* CPCC 401041, only the relative positions of four genes (*cox2*, *cob*, *atp6*, *nad1*) differed (Fig. S3). In terms of gene arrangement, *A. psychrotrophicus* CPCC 401038 and *A. pellizariae* UFMGCB 12416 exhibited greater similarity. Compared to *A. psychrotrophicus* CPCC 401038, the relative positions of tRNA genes in the mitochondrial genome of *T. microsporus* CPCC 401041 remained largely unchanged, except for minor variations in the number of tRNA genes. Across the three mitochondrial genomes centered on *atp6*, positional changes in two gene boxes (Box 1 and Box 2) were observed, with the relative positions of the *cox2* and *cob* genes reversed (Fig. 4). Furthermore, the relative positions of the two rRNA genes remained unchanged between the two mitochondrial genomes. These findings suggested that the gene order in the mitochondrial genomes of the genera *Antarctomyces* and *Thelebolus* was relatively conserved. A comparison of the gene order in the mitochondrial genomes of psychrophilic, psychrotrophic, and mesophilic fungi revealed numerous rearrangements in their PCGs and rRNA genes, occurring in a highly variable order (Fig. S4).

**Fig. 5:**
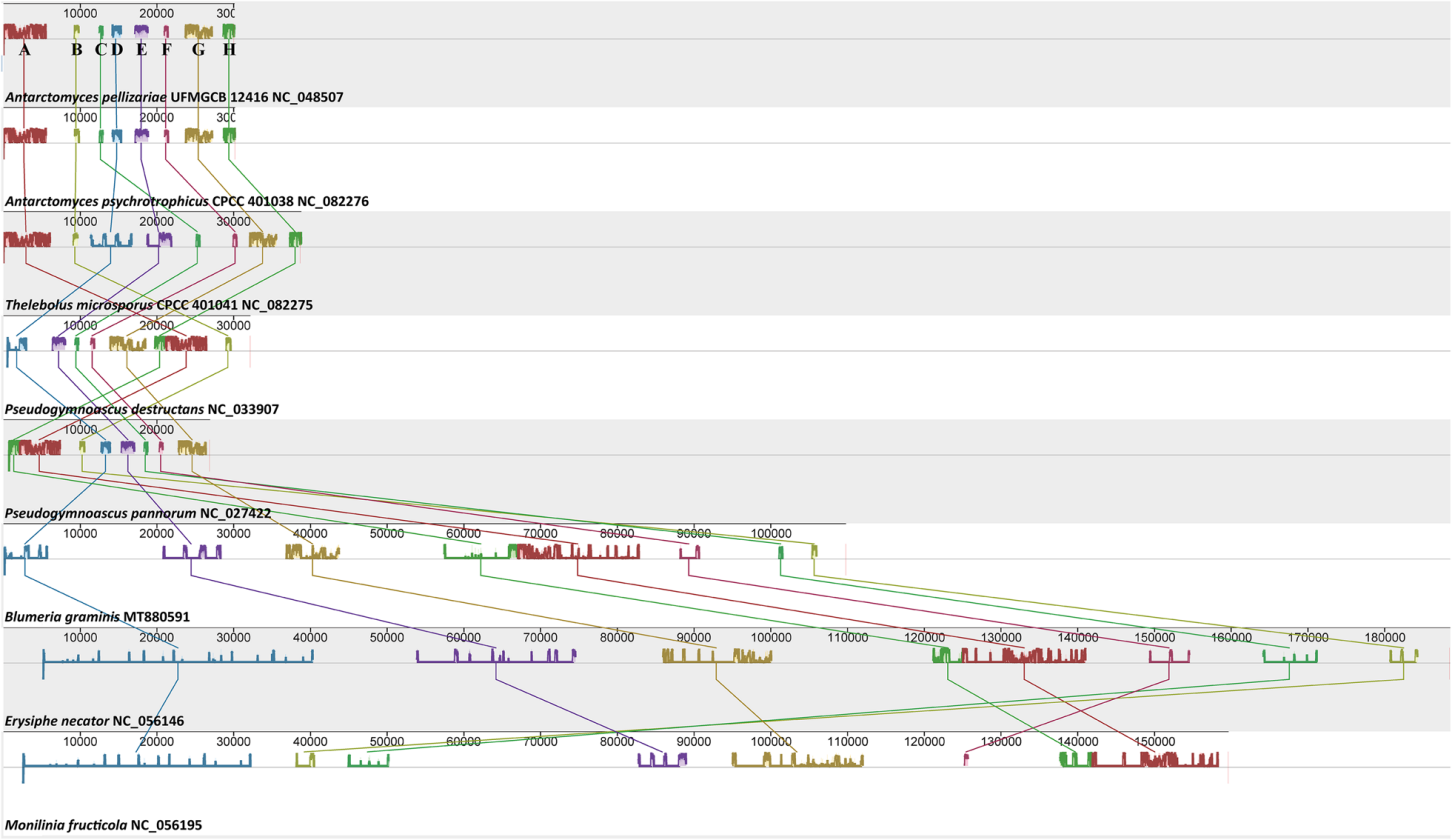
Collinearity analysis of the eight mitochondrial genomes of psychrophilic, psychrotrophic, and mesophilic fungi

Collinearity analysis of the eight mitochondrial genomes identified a total of eight homologous regions (Fig. 5). These homologous regions are longer in mesophilic fungi, likely as a result of extensive rearrangements in the genomes. Additionally, the relative positions of these homologous regions were more consistent in psychrophilic fungi, and the three homologous regions A, B, and H exhibited co-movement in psychrotrophic fungi.

### Genetic distance and evolutionary rates of genes

**Fig. 6:**
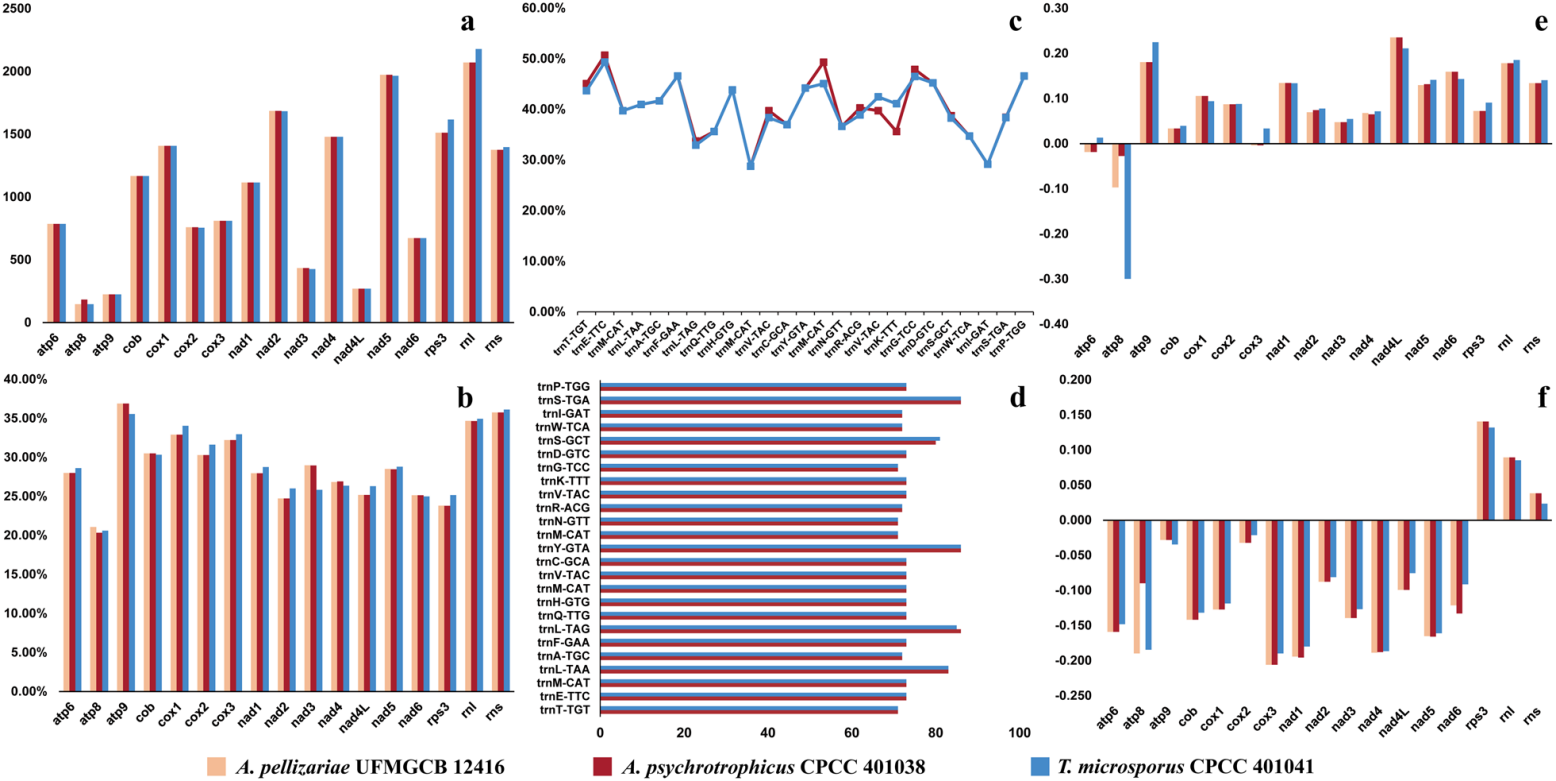
Differences in length and base composition among 15 PCGs, two rRNA genes, and 25 tRNA genes across three mitogenomes. **a** length variation of the PCGs and rRNA genes; **b** GC content of the PCGs and rRNA genes; **c** GC content of shared tRNA genes; **d** lengths of shared tRNA genes; **e** GC skew; **f** AT skew

Among the 15 PCGs in the mitochondrial genomes of *A. pellizariae* UFMGCB 12416, *A. psychrotrophicus* CPCC 401038, and *T. microsporus* CPCC 401041, *rps3* showed the greatest length difference, with a variation of 105 bp. The varying GC contents of the PCGs in three mitochondrial genomes suggested that the bases of each core PCG underwent frequent changes in *A. psychrotrophicus* CPCC 401038 and *T. microsporus* CPCC 401041 (Fig. 6). Among the 15 PCGs in three mitochondrial genomes, *atp9* had the highest GC content, whereas *atp8* had the lowest. The GC skew was more variable in mitochondrial genomes, with a negative skew observed for the *atp8* gene in all three genomes, and for *atp6* and *cox3* in the two genomes of *A. pellizariae* UFMGCB 12416 and *A. psychrotrophicus* CPCC 401038. All PCGs in these three mitochondrial genomes exhibited negative AT skews, except for the *rps3* gene. Significant differences were observed among the three genomes, particularly in the genes *atp8*, *nad6*, and *nad4L*. Additionally, the lengths of the tRNA genes shared between the mitochondrial genomes of *A. psychrotrophicus* CPCC 401038 and *T. microsporus* CPCC 401041 were nearly identical, with only *trnL-TAG* and *trnS-GCT* differing by one base. All tRNA genes had GC levels ranging from 28.77% to 50.68%, with *trnM-CAT* having the lowest and *trnE-TTC* the highest. The two mitochondrial genomes shared 25 tRNA genes, but only nine of them had identical GC content. *TrnK-TTT* exhibited the highest GC content.

The remaining 14 PCGs were analyzed for correlations between genetic variation and growth temperature, as *atp9* was absent from the mitochondrial genomes of some fungi. Several genes in mesophilic fungi, including *atp8*, *cox2*, *cox3*, *nad5*, and *rps3*, exhibited higher GC content, with *rps3* showing more pronounced expression. When considering the 14 PCGs and 2 rRNA genes together, most GC contents in psychrophilic fungi were lower, and the lengths of individual genes were more consistent. Additionally, the GC skew of individual genes in psychrophilic, psychrotrophic, and mesophilic fungi was negative, while the AT skew of the *rps3* gene showed both positive and negative values.

**Fig. 7:**
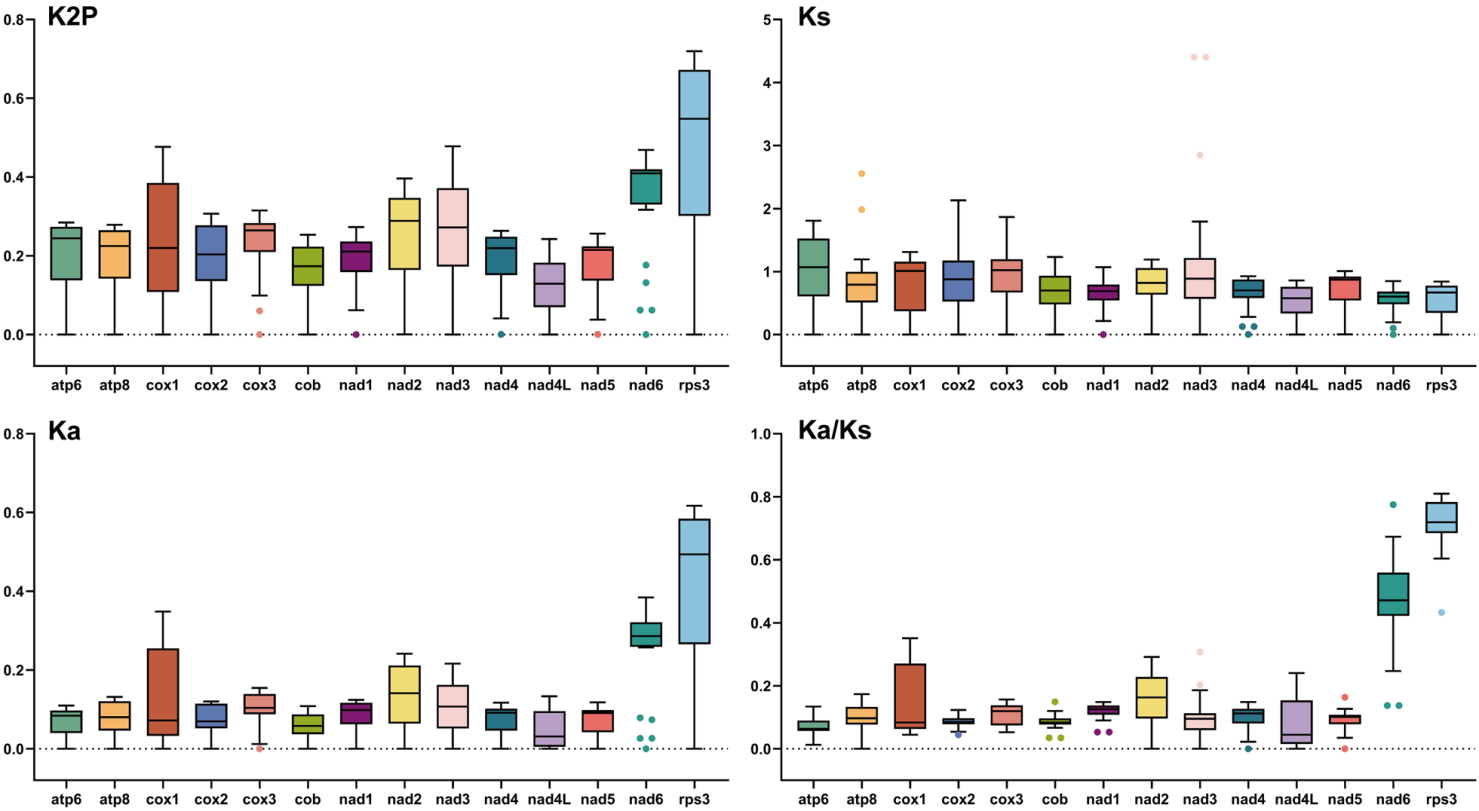
Genetic analysis of 14 PCGs conserved across eight mitogenomes. K2P: the Kimura-2-parameter distance; Ka: the mean number of nonsynonymous substitutions per nonsynonymous site; Ks: the mean number of synonymous substitutions per synonymous site

Among the 14 PCGs in these eight mitochondrial genomes, *rps3* exhibited the highest average K2P genetic distance, followed by *nad6* and *nad3*, suggesting that these genes have become more variable (Fig. 7). In contrast, *nad4L* had the lowest average K2P genetic distance among these eight species, followed by *cob* and *nad5*, indicating these genes are more evolutionarily conserved. When calculating Ks and Ka, *nad4L* had the smallest values for both, and its Ka/Ks value was only 0.009 higher than that of *atp6* among the 14 PCGs. Additionally, *rps3* had the highest Ka, and *nad3* had the largest Ks. All Ka/Ks values of these 14 PCGs were less than 1, indicating they are all under purifying selection, with *rps3* having the highest Ka/Ks value of 0.716.

### Mitochondrial phylogeny of *Leotiomycetes*

**Fig. 8:**
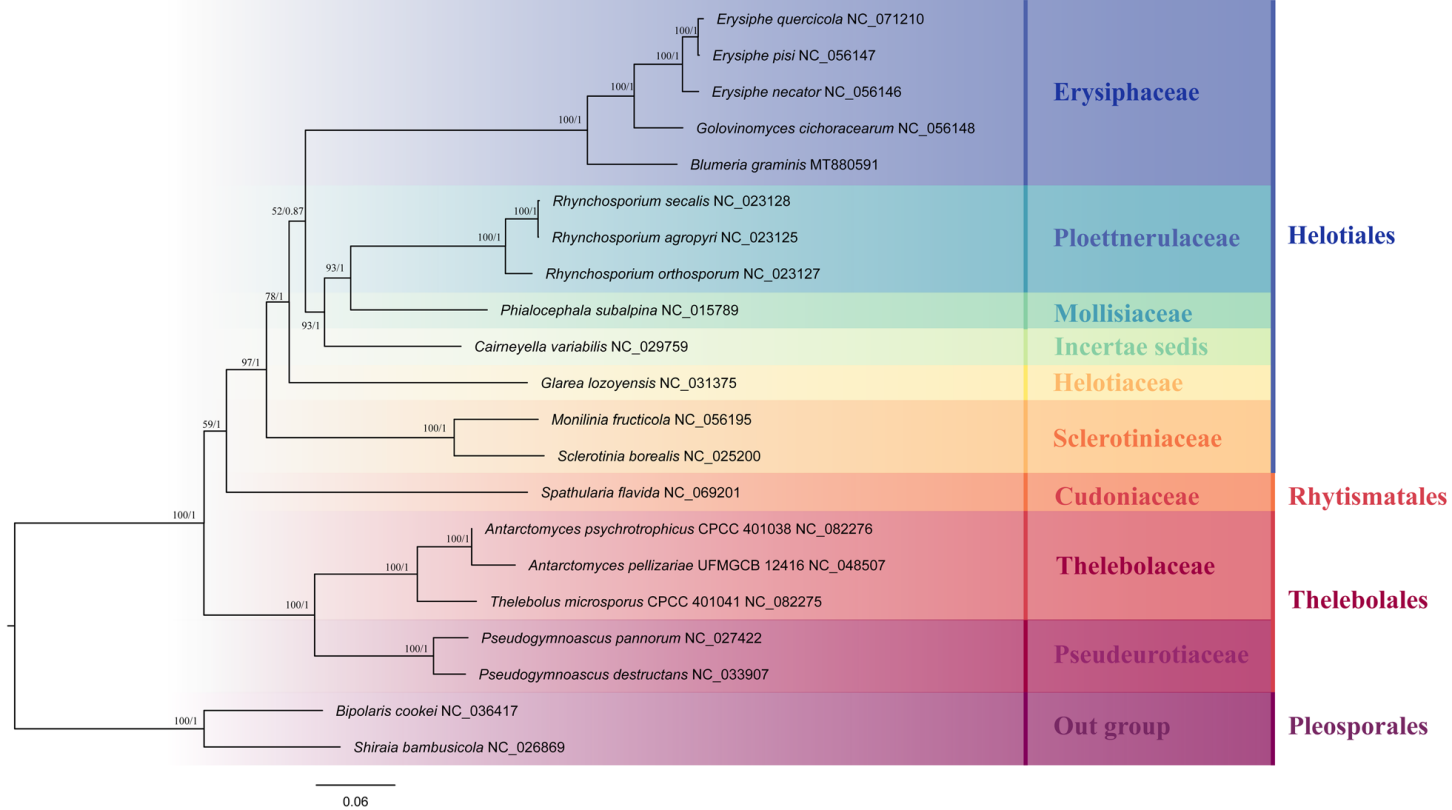
Phylogenetic tree based on mitochondrial gene sets. Bootstrap values (BS) are shown before the slash on the branch, and Bayesian posterior probabilities are shown after the slash

A well-supported phylogenetic tree was generated using concatenated mitochondrial gene sets (14 PCGs) through analyses based on Bayesian inference (BI) and maximum likelihood (ML) methods (Fig. 8). Based on phylogenetic analysis, 21 *Leotiomycetes* species can be divided into four main evolutionary branches: the order *Helotiales*, which includes the families *Erysiphaceae*, *Helotiaceae*, *Mollisiaceae*, *Ploettnerulaceae*, and *Sclerotiniaceae*; the order *Pleosporales*, which includes the families *Pleosporaceae* and *Shiraiaceae*; the order *Rhytismatales*, which includes the family *Cudoniaceae*; and the order *Thelebolales*, which includes the families *Thelebolaceae* and *Pseudeurotiaceae*. Within the family *Thelebolaceae*, *A. psychrotrophicus* CPCC 401038 and *T. microsporus* CPCC 401041 clustered together on a branch with *A. pellizariae* UFMGCB 12416. Therefore, the analysis suggests that the combined mitochondrial gene dataset serves as an appropriate and reliable molecular marker for studying phylogenetic relationships among species.

## Discussion

In this study, the mitochondrial genomes of *A. psychrotrophicus* CPCC 401038 and *T. microsporus* CPCC 401041 differed by 8633 bp, despite having nearly identical GC content. The expansion in intronic and intergenic regions was considered a major factor contributing to the larger genome size of *T. microsporus* CPCC 401041. These findings are consistent with previous studies indicating that introns are crucial for altering fungal mitochondrial size (Li et al. 2018a, b, c; Ye et al. 2020). The dynamics of introns, regarded as mobile genetic components in fungal mitochondria, significantly affect the size and arrangement of fungal mitochondria (Hamari et al. 2005; Repar et al. 2017; Sandor et al. 2018). Except for one tRNA gene, all genes in *T. microsporus* CPCC 401041 are located on a single strand of the mitochondrial genome. This finding is consistent with a previous study showing that most mitochondrial genes are typically located on the same strand in ascomycete mitochondria (Aguileta et al. 2014). By comparing the mitochondrial genomic characteristics of psychrophilic, psychrotrophic, and mesophilic fungi, we can conclude that mesophilic fungi have larger mitochondrial genomes, higher GC content, more introns, and longer intergenic regions. The high GC content likely enhances mitochondrial genome stability under high-temperature conditions, while genome expansion provides additional genetic information. These factors contribute to a better understanding of fungal adaptability to different growth temperatures at the mitochondrial genome level.

Compared to primitive nuclear genomes, in which mitochondrial genes were transferred to eukaryotic nuclear genomes during evolution, mitochondrial genomes have diverged significantly from eukaryotic nuclear genomes since the acquisition of eukaryotic mitochondrial genomes from ancestral endosymbiotic bacteria (Chen et al. 2014; Thielsch et al. 2017). This phenomenon highlights several benefits conferred by this evolutionary process (Adams et al. 2003). A small subset of mitochondrial genes, including two rRNA genes, 5–35 tRNA genes, and a set of key PCGs for energy metabolism, has survived (Allen 2015; Wang et al. 2020a, b). These preserved genes significantly influence the control of mitochondrial activity and cell homeostasis (Allen 2015; Björkholm et al. 2015). Fifteen PCGs were identified in both mitochondrial genomes: *atp6*, *atp8*, *atp9*, *cob*, *cox1*, *cox2*, *cox3*, *rps3*, *nad1*, *nad2*, *nad3*, *nad4*, *nad4L*, *nad5*, and *nad6*. The AT skew and GC skew of these genes differed between the two species, highlighting their genetic differentiation. Furthermore, the two species in this study have different rRNA and tRNA gene lengths and base compositions. Previous studies indicates that base mutations in mitochondrial tRNAs can impact protein synthesis (Ding et al. 2019; Lin et al. 2019). However, further research is needed to determine how rRNA and tRNA variations affect fungi adapted in polar conditions. Comparing the mitochondrial genes of fungi growing at different temperatures showed that mesophilic fungi had a higher GC content of mitochondrial genes, which favors stabilization under high-temperature conditions. Additionally, comparison of the genetic distances and Ka/Ks of the 14 PCGs shared by the eight fungi showed that *nad4L* was more conserved, while *nad3* and *rps3* were more variable.

The organization of mitochondrial DNA could serve as a valuable reference for assessing phylogenetic relationships and genetic connections among species (Li et al. 2018a, b, c; Li et al. 2018a, b, c; Wang et al. 2020a, b). The gene arrangement in the mitochondrial genomes of *A. psychrotrophicus* CPCC 401038 and *T. microsporus* CPCC 401041 was less variable, with only a few tRNA gene additions or deletions and rearrangements of three core genes (*cox2*, *cob*, *nad1*). When analyzing the differences in genes within the mitochondrial genomes, the organization of *A. psychrotrophicus* CPCC 401038 is more comparable to that of *A. pellizariae* UFMGCB 12416. This finding suggests that mitochondrial gene differences can aid in distinguishing species from different genera. By comparing the gene order and homology regions in the mitochondrial genomes of psychrophilic, psychrotrophic, and mesophilic fungi, we discovered that numerous rearrangements have occurred, contributing to their evolutionary processes. Mesophilic fungi also have longer homologous regions, primarily due to the insertion of additional introns into genes in these regions, resulting in a wider range of homologous region sizes.

The variation in the location of homologous regions across fungi also suggests that the mitochondrial genome can provide novel approaches to the study of species classification and phylogenetic relationships. Two-thirds of the named fungal species belong to the phylum *Ascomycota*, which includes numerous economically important species, as well as a wide range of pathogens, decomposers, and symbionts (Díaz-Escandón et al. 2022; Spatafora et al. 2017). This highlights the importance of accurate taxonomy and identification in better exploiting or controlling them. The classification of fungi currently relies on nuclear genomic and molecular markers (James et al. 2006; Spatafora et al. 2016). However, mitochondrial genomes are more accessible and contain more genetic information, making them highly promising for the classification and identification of ascomycetes (Li et al. 2022). Therefore, molecular markers should be used in conjunction with phylogenetic and taxonomic investigations of fungi, with mitochondrial genomes serving as a valuable addition (Chen et al. 2019). In this study, data from the 14 common protein-coding genes used to construct the phylogenetic tree can serve as reliable molecular markers. In this study, the data from the 14 common protein-coding genes can be used as reliable molecular markers to build the phylogenetic tree. The two species, *A. psychrotrophicus* CPCC 401038 and *T. microsporus* CPCC 401041, are most closely related to *A.* pellizariae (de Menezes et al. 2017). However, the majority of single-gene phylogenetic trees show distinct evolutionary branches, and some even fail to distinguish between various classes of fungi, presumably because individual genes do not convey enough evolutionary information (Wang et al. 2017).

In previous studies, the only mitochondrial genome of the family *Thelebolaceae* available in the NCBI database is that of *Antarctomyces pellizariae*, a circular genome of 30,121 bp that has not been extensively characterized. This study is the first to systematically analyze the mitochondrial characteristics of *Antarctomyces psychrotrophicus* and *Thelebolus microsporus*, and to compare their mitochondrial genomes within the family *Thelebolaceae*. This study is significant for the taxonomy of the family *Thelebolaceae*, as it fills a gap in the mitochondrial genome data avaible for this group. A detailed mitogenomic comparison within order *Thelebolales* and family *Thelebolaceae* could provide valuable insights into their evolution and ecology. However, due to the lack of mitochondrial genomic data for other species within *Thelebolales*, broader analysis is limited. Future studies should focus on sequencing and analyzing mitochondrial genomes from additional Thelebolales species to better understand their evolutionary relationships and ecological adaptations.

Fungal species with varying temperature adaptations were compared at the mitochondrial genome level to identify the similarities and differences, providing a basis for studying the cold-adaptation mechanisms of psychrophilic fungi. Similar mitochondrial adaptations might also be observed in fungi from other extreme conditions, such as high salinity, extreme pH, or high temperatures. Further comparative studies of diverse fungi are essential to understand the extent and specificity of these mitochondrial traits. Such studies could reveal common evolutionary strategies that enable fungi to thrive under extreme conditions, providing deeper insights into fungal adaptation and versatility.

## Conclusion

This study presents the first comparative analysis of mitochondrial genomes within the family *Thelebolaceae*, focusing on two newly assembled genomes from the Antarctic genera *Antarctomyces* and *Thelebolus*, as well as two psychrotrophic fungi and three mesophilic fungi from outside the family *Thelebolaceae*. We found that mesophilic fungi exhibited higher GC content and a larger proportion of intergenic and intronic regions compared to their psychrotrophic and psychrophilic counterparts. Additionally, we observed significant variability in the GC content across the protein-coding genes (PCGs) of these eight genomes, with psychrophilic fungi exhibiting the lowest GC content. Furthermore, gene comparison revealed that the two mitochondrial genomes from the same genus (*Antarctomyces*) were more similar. Different evolutionary rates were evident among the PCGs of psychrophilic, psychrotrophic, and mesophilic fungi, with *nad4L* being the most conserved and *rps3* the most divergent. All PCGs were under purifying selection, and considerable rearrangements were observed in the PCGs and rRNA genes, indicating a highly variable gene order. These findings enhance our understanding of the extremophilic adaptations and evolutionary dynamics within the family *Thelebolaceae* in Antarctica, highlighting how varying environmental temperatures influence fungal mitochondrial genomic structure and adaptation.

## Supplementary Information

Supplementary Material 1.

## Data Availability

All data generated or analyzed during this study are included in this published article.

## Abbreviations

CPCC: China Pharmaceutical Culture Collection
NCBI: National Center for Biotechnology Information
PRICE: Paired-Read Iterative Contig Extension
PCG: Protein-coding gene
K2P: Kimura-2-parameter
Ks: Synonymous substitution rates
Ka: Nonsynonymous substitution rates
BI: Bayesian inference
BS: Bootstrap support
ML: Maximum likelihood
tRNA: Transfer RNA
rRNA: Ribosomal RNA
rnl: Large subunit ribosomal RNA
rns: Small subunit ribosomal RNA
