# Supplementary material for: Comparative mitochondrial genomics of Thelebolaceae in Antarctica: insights into their extremophilic adaptations and evolutionary dynamics
Source: IMA Fungus. 2024 Oct 30;15:33. doi: 10.1186/s43008-024-00164-7 (PMC11523780; doi:10.1186/s43008-024-00164-7)
Supplement: Supplementary file 1 — Supplementary Material 1. [file 43008_2024_164_MOESM1_ESM.docx]

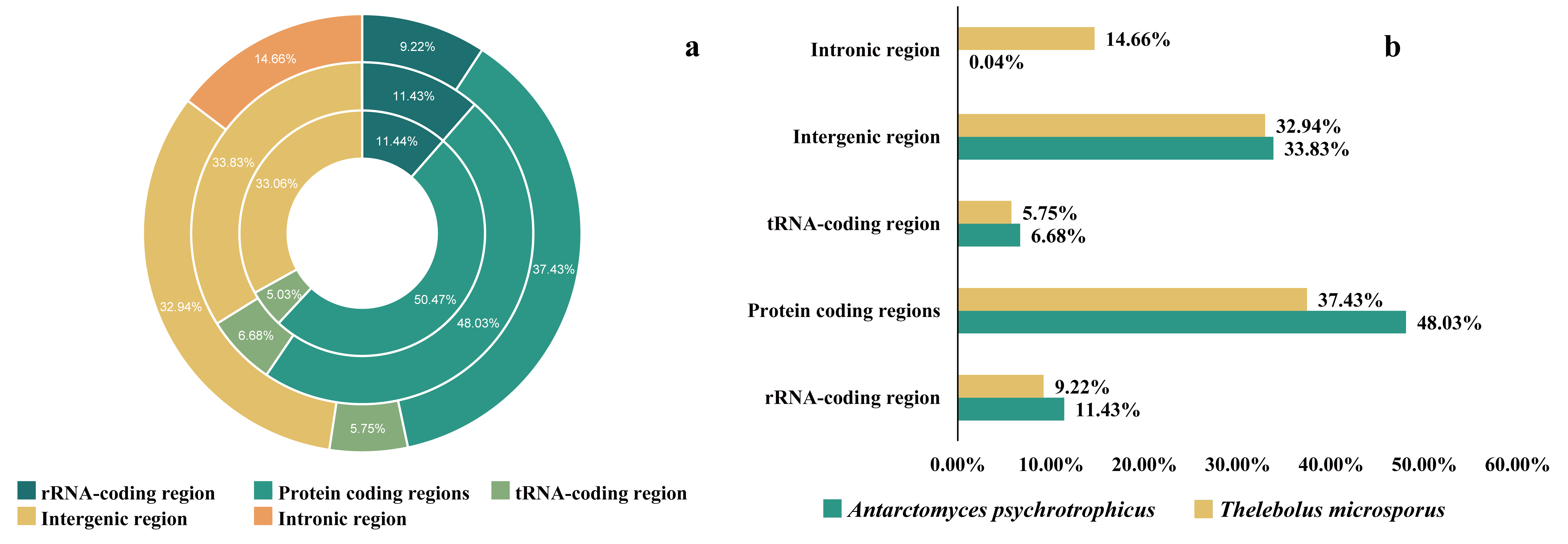
**Fig. S1** **a** Rings showing the proportional composition of each region in the three mitochondrial genomes. The inner to outer circles represent *A. pellizariae* UFMGCB 12416, *A. psychrotrophicus* CPCC 401038, and *T. microsporus* CPCC 401041. **b** Contribution of different genetic regions to the expansion of the mitochondrial genomes of *A. psychrotrophicus* CPCC 401038, and *T. microsporus* CPCC 401041.


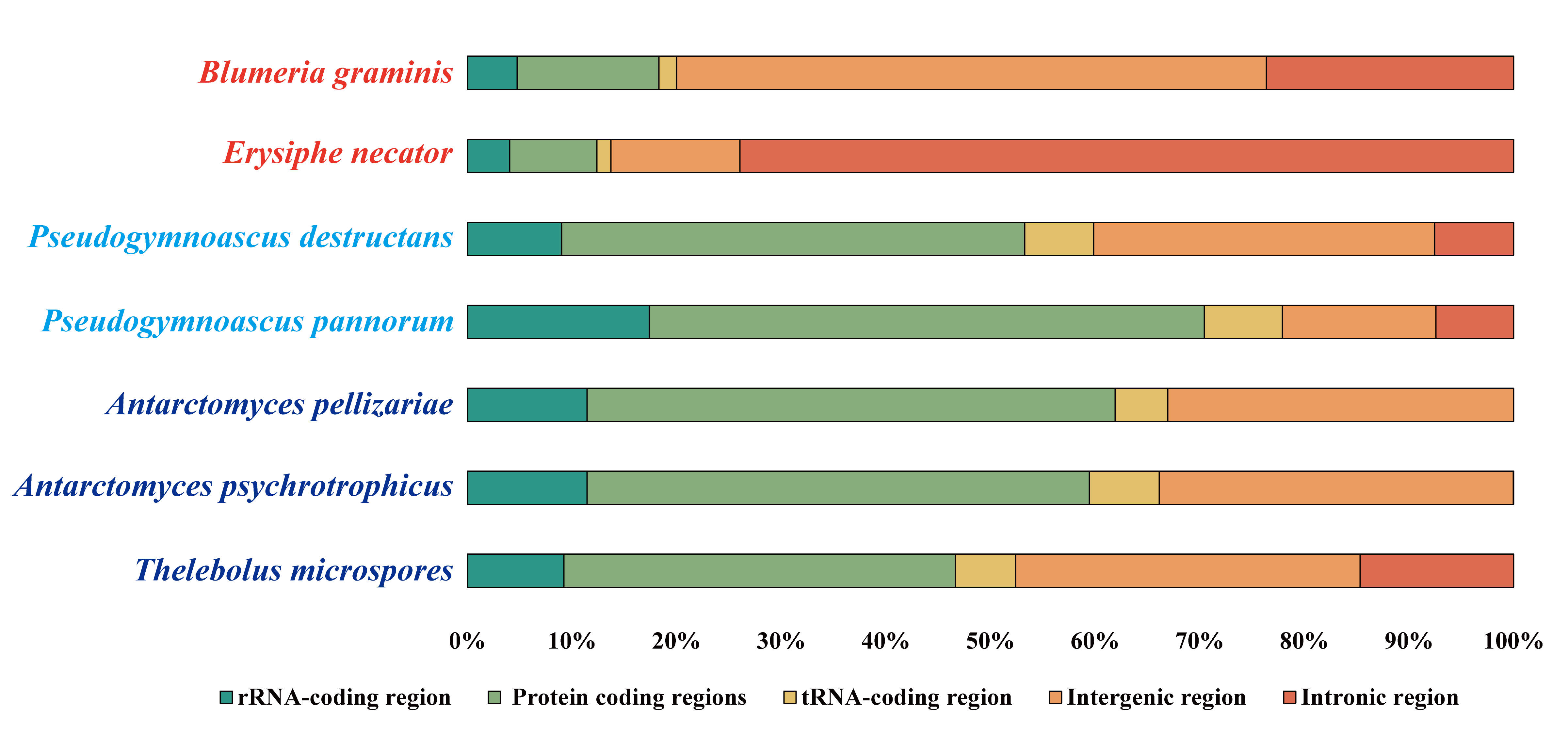


**Fig. S2** Proportion of each region in the mitochondrial genomes of psychrophilic, psychrotrophic, and mesophilic fungi. *Monilinia fructicola* is excluded due to incomplete information regarding the intronic and intergenic regions in its mitochondrial genome annotation.


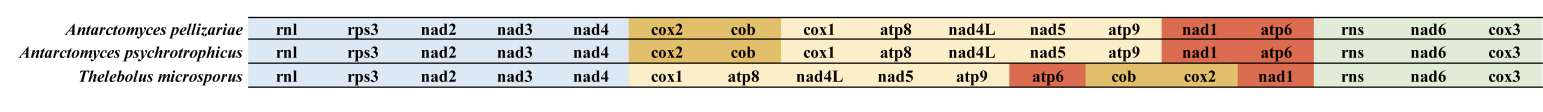


**Fig. S3** Comparison of the arrangement order of PCGs and rRNA genes in the mitochondrial genomes of *A. pellizariae* UFMGCB 12416, *A. psychrotrophicus* CPCC 401038, and *T. microsporus* CPCC 401041. Different colored blocks are used to distinguish gene rearrangements.


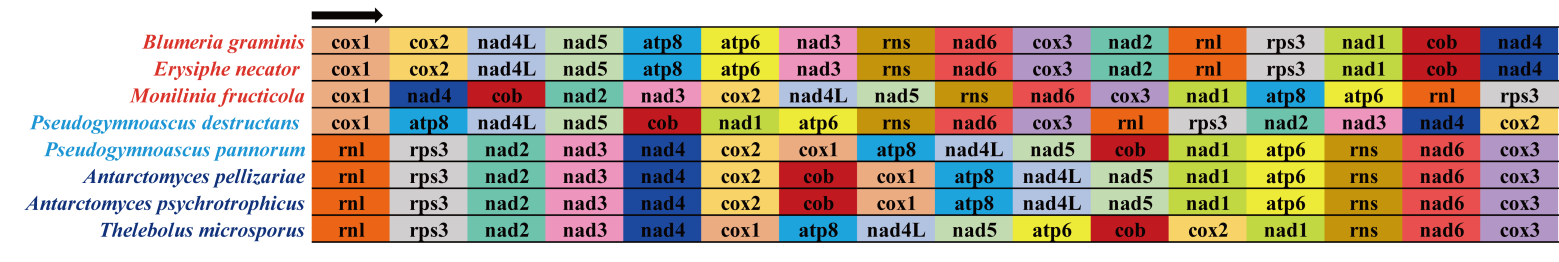


**Fig. S4** Comparison of the order of PCGs and rRNA genes in the mitochondrial genomes of psychrophilic, psychrotrophic, and mesophilic fungi.
